# Using Artificial Neural Network to Discriminate Parkinson’s Disease from Other Parkinsonisms by Focusing on Putamen of Dopamine Transporter SPECT Images

**DOI:** 10.3390/biomedicines9010012

**Published:** 2020-12-24

**Authors:** Chung-Yao Chien, Szu-Wei Hsu, Tsung-Lin Lee, Pi-Shan Sung, Chou-Ching Lin

**Affiliations:** 1 Department of Biomedical Engineering, National Cheng Kung University, Tainan 704, Taiwan; p88041075@ncku.edu.tw; 2 Department of Neurology, National Cheng Kung University Hospital, College of Medicine, National Cheng Kung University, Tainan 704, Taiwan; c2481023@hotmail.com (T.-L.L.); pishansung@gmail.com (P.-S.S.); 3 Department of Nuclear Medicine, National Cheng Kung University Hospital, College of Medicine, National Cheng Kung University, Tainan 704, Taiwan; i54921514@gmail.com

**Keywords:** artificial neural network, deep learning, Parkinson’s disease, atypical parkinsonian syndrome, dopamine transporter SPECT

## Abstract

Background: The challenge of differentiating, at an early stage, Parkinson’s disease from parkinsonism caused by other disorders remains unsolved. We proposed using an artificial neural network (ANN) to process images of dopamine transporter single-photon emission computed tomography (DAT-SPECT). Methods: Abnormal DAT-SPECT images of subjects with Parkinson’s disease and parkinsonism caused by other disorders were divided into training and test sets. Striatal regions of the images were segmented by using an active contour model and were used as the data to perform transfer learning on a pre-trained ANN to discriminate Parkinson’s disease from parkinsonism caused by other disorders. A support vector machine trained using parameters of semi-quantitative measurements including specific binding ratio and asymmetry index was used for comparison. Results: The predictive accuracy of the ANN classifier (86%) was higher than that of the support vector machine classifier (68%). The sensitivity and specificity of the ANN classifier in predicting Parkinson’s disease were 81.8% and 88.6%, respectively. Conclusions: The ANN classifier outperformed classical biomarkers in differentiating Parkinson’s disease from parkinsonism caused by other disorders. This classifier can be readily included into standalone computer software for clinical application.

## 1. Introduction

Disease-modifying therapies including target therapy are under development to treat Parkinson’s disease (PD). According to the targeted pathogenesis, some treatment strategies focus on the very initial phase of the disease [1,2]. However, early in the disease progress, PD and parkinsonism caused by other disorders, including atypical parkinsonian syndromes share similar clinical features because the hallmarks of PD or other parkinsonism may not have emerged [3,4]. To date, the diagnosis of PD is solely based on clinical diagnostic criteria and gene tests. However, it takes time to fulfil these clinical diagnostic criteria, and only less than 5% of all PD patients have known causative genes [1,5]. Therefore, new diagnostic tools aiding efficient screening are required to address this unmet need.

Clinicopathological studies based on brain bank material have shown that clinicians diagnose PD incorrectly in about 25% of patients. One of the most common reasons for misdiagnosis was atypical parkinsonian syndromes [6]. To differentiate PD from other forms of parkinsonism, the guidelines of the European Federation of Neurological Societies suggest transcranial sonography of the mesencephalic brainstem. In clinical practice, proper evaluation of the substantial nigra depends on experienced technicians and investigators, and also on the quality of the temporal bone window. Structural magnetic resonance imaging (MRI) reveals typical signs of Parkinson-plus syndromes only in the middle or later course of the diseases. Many types of advanced MRI techniques such as voxel-wise analysis [7], diffusion [8,9], susceptibility [10,11], neuromelanin [12], and functional imaging have been evaluated, however their overall sensitivity and specificity have been insufficient to meet the clinical demand. 18F-fluorodeoxyglucose positron emission tomography (FDG-PET) is an imaging modality that has a prediction accuracy above 90% [13,14]. Due to the long procedure time, the influence of the subject’s blood glucose status, cost-effectiveness, and usage of diagnostic template images, to date, FDG-PET has not been recommended in clinical practice. Moreover, other clinical diagnostic modalities such as 123I-metaiodobenzylguanidine (MIBG) myocardial scintigraphy and olfactory testing have been reported to achieve a higher specificity of up to 80% when compared with gold-standard clinical or clinicopathologic diagnoses in differentiating PD from other parkinsonisms [3]. However, several olfactory test studies have reported a sensitivity ranging from 61–77% [3,5,15,16], and when MIBG myocardial scintigraphy was used prospectively in general parkinsonian cases, the accuracy was somewhat limited [17].

An abnormal dopamine transporter single-photon emission computed tomography (DAT-SPECT) image reflects the dysfunction of striatal neurons, and its discrimination of PD or not PD relies on clinical information and other structural images. However, in daily clinical scenarios discriminative information is not always obtainable. To classify parkinsonism based on DAT-SPECT images, advanced engineering techniques with semi-quantitative analysis have been applied [18]. In addition, images or signals from striatal regions (SRs) alone can provide adequate differentiating information [19]. One research group differentiated degenerative parkinsonism using a computer-aided automatic algorithm and SR and whole-brain uptake patterns. Both were shown to have adequate specificity (84–90%), however the whole-brain uptake pattern demonstrated lower sensitivity [20]. Another study group discovered that in voxel-based analysis of DAT-SPECT images, SR alone could differentiate PD from dementia with Lewy bodies (DLB), while regions outside SRs were not contributory [21].

Machine learning and artificial neural networks (ANNs) have developed rapidly and been applied in clinical settings [22]. Recently, Vaccaro et al. demonstrated that a careful analysis of neuropsychological deficits through a machine-learning approach successfully discriminated PD and progressive supranuclear palsy [23]. An ANN application on DAT-SPECT images reported a classification accuracy of up to 90% in identifying PD with a mean Hoehn and Yahr (H&Y) stage of 1.6 from healthy controls [24], a great leap from the 80% achieved with conventional or semi-quantitative analysis [24,25]. Thus, in this study, we combined appropriate segmentation of SR images derived from DAT-SPECT with a widely-used pre-trained neural network for computer-vision to investigate the efficiency of this integrated method in identification of PD from parkinsonism caused by other disorders.

## 2. Material and Methods

### 2.1. Subjects

Ethical review and approval were waived for this study, due to collection, analysis and publication of the retrospectively obtained and anonymized data for this non-interventional study. As a retrospective study evaluating SPECT images performed in the diagnostic setting without disclosing any personal information of the patients, the need for written consent was waived.

#### 2.1.1. First Set of Images for ANN Training and Validation

**Figure 1:**
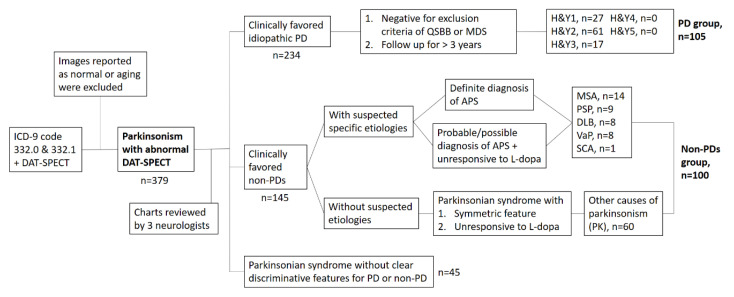
The flow chart of subject selection for artificial neural network (ANN)-classifier training. The cases with drug-induced parkinsonism which reported as normal DAT-SPECT were excluded. ICD, international classification diagnosis; DAT-SPECT, dopamine transporter single-photon emission computed tomography; PD, Parkinson’s disease; APS, atypical parkinsonian syndrome; QSBB, Queen Square Brain Bank; MDS, movement disorder society; H&Y, Hoehn and Yahr stage; MSA, multiple system atrophy; PSP, progressive supranuclear palsy; DLB, dementia with Lewy bodies; VaP, vascular parkinsonism; SCA, spinocerebellar ataxia.

Medical charts of subjects with parkinsonian syndromes (ICD-9 coded 332.0 and 332.1) who received DAT-SPECT imaging (99mTc-TRODAT-1-SPECT) from 2017 to 2019 at the outpatient clinic performed by three neurologists specializing in movement disorders were retrospectively reviewed. The initial number of collected subjects was 518. The images reported as normal or aging-related were firstly excluded. The remaining 379 patients (234 subjects with clinically-favored idiopathic PD and 145 subjects with clinically-favored non-PD) were then assigned into two groups: those with PD and those with parkinsonism caused by other disorders (non-PDs), according to the following criteria. In the PD group, in order to establish higher sensitivity and specificity (>90%) of “ground truth” images, we followed the Queen Square Brain Bank (QSBB) criteria to exclude those with a history of stroke or exposure to neuroleptic agents. Finally, 105 cases who had been regularly followed up for more than three years were classified into the PD group. In the non-PD group, cases with drug-induced parkinsonism were excluded, and 100 cases with a diagnosis of possible or probable Parkinson-plus syndromes (such as multiple system atrophy or progressive supranuclear palsy), DLB, vascular parkinsonism, or other causes of parkinsonism characterized by parkinsonian syndromes with symmetrical features and unresponsive to L-dopa treatment were selected (Figure 1). Finally, a total of 205 images were used to train the ANN as a classifier through randomly splitting these images into 90% for training and 10% for validation.

#### 2.1.2. Second Set of Images for Testing the ANN Classifier

To test the performance of the trained ANN classifier, a second dataset of DAT-SPECT images performed from January to March 2020 of cases with a diagnosis of parkinsonian syndrome (n = 57) was obtained. Cases with a history of unilateral onset of parkinsonian symptoms and adequate responsiveness to levodopa treatment, but who did not meet the QSBB exclusion criteria were defined as having PD. Those with prominent red flags such as bilateral onset of symptoms and unresponsive to levodopa treatment, or who met the QSBB exclusion criteria such as early cognitive impairment, cerebellar signs, or with structural imaging suggesting vascular parkinsonism or hydrocephalus were defined as having parkinsonism caused by other disorders (non-PDs).

### 2.2. Image Processing

#### 2.2.1. Image Pre-Processing

First, a mask to remove scalp uptake was applied to all images. The intensity of images was then normalized by contrast stretching. To select the region of interest (ROI), i.e., the SR, an active contour model was applied [26]. The physician first selected an ROI using the same procedure as in the conventional method for calculating striatal/occipital ratio, and the active contour model automatically adjusted the outline of the ROI [27] to minimize the summarized values contributed by both inside and outside of the ROI, and a fitted ROI was then segmented out for the next step. This method also minimized selection bias and physician inconsistency. We also kept the images before segmentation for further comparison.

#### 2.2.2. Binary Classification by ANN

**Figure 2:**
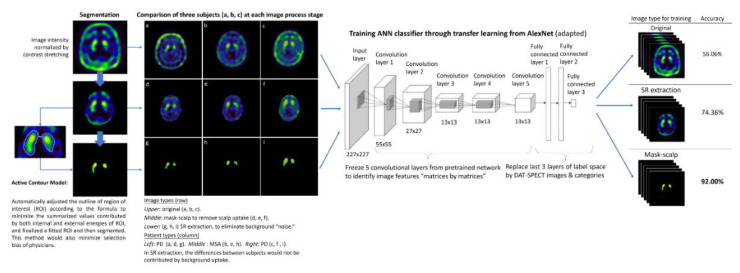
Workflow of image preprocessing, SR segmentation, and ANN classifier training. The ANN classifier was trained by different types of images (original, whole brain, and segmented SR). The SR segmentation demonstrated higher accuracy than the other two types of images.

The segmented SR images were fed into the ANN training process for classification. We applied the method of transfer learning to a pretrained network from an open source. AlexNet is a standard model for image classification through deep learning that has been widely applied to medical images. It is composed of five convolutional layers and three fully-connected layers. We froze the parameters of convolutional layers for basic feature extraction. In the last three fully-connected layers, we replaced the label space with our image categories. This trained ANN classifier was first validated using the validation data targeting an accuracy > 90%, and then re-confirmed using the independent test dataset. The results of training/validation and test dataset were presented by calculating the area under the receiver operating characteristic curve (AUROC). For comparison, we also trained another ANN classifier using images of the whole brain without segmentation (Figure 2).

#### 2.2.3. Semi-Quantitative Measurements and Machine-Learning Classification

Two indicators were evaluated—specific binding ratio (SBR), which was calculated as ((SR-occipital)/occipital) and asymmetry index (ASI), which was calculated as ((2 |SRleft − SRright|)/(SRleft + SRright)). Classification of the PD and non-PD groups was attempted using SBR and ASI with machine-learning approaches including linear discrimination, support vector machine (SVM) with quadratic, cubic, and Gaussian kernel methods, with or without primary component analysis (PCA) from the classification learner toolbox of Matlab 2018b (MathWorks, Natick, Massachusetts). The SVM handled both linear and nonlinear classification. In linear models, the SVM attempted to define the largest margin between the points on either side of the decision line, whereas in non-linear models, a hyperplane approach was applied for binary classification of the dataset.

Details of the DAT-SPECT scanning protocol and imaging data acquisition are described in Appendix A. Statistical analyses were performed using SPSS software (SPSS Statistics for Windows, version 17.0, SPSS Inc., Chicago, IL, USA).

#### 2.2.4. Class-Activation Mapping to Visually Explain the ANN Classifier

Computer-vision examines images in matrices using a matrix method and convolutes them into complicated features which are usually meaningless to the human eye. These features are not regarded as being biomarkers and are hardly correlated to clinical facts. One way to visualize computer-vision is through class-activation mapping (CAM), which produces “visual explanations” from an ANN using parts of the image that weigh most while performing classification. CAM has been widely applied in deep learning methods of medical imaging [28], and we used it in this study to visually explain the results from the ANN classifier.

All image processing and ANN procedures were implemented in Matlab 2018b (MathWorks, Natick, MA, USA).

## 3. Results

### 3.1. Demographic Characteristics

The clinical characteristics of the patient groups are summarized in Table 1. There were no significant differences in age or gender (for training/validation set, *p* = 0.44 and for test set, *p* = 0.91). For the training/validation dataset, there were 105 subjects in the PD group with an average H&Y stage of 1.93 (median H&Y stage 2). The non-PD group (100 subjects) included 23 cases with Parkinson-plus syndrome, 8 cases with DLB, 8 cases with vascular parkinsonism, 1 case with spinocerebellar ataxia, and 60 cases with other forms of parkinsonism. For the test dataset, there were 22 subjects in the PD group, with an average H&Y stage of 1.95 (median H&Y stage 2), and 35 subjects in the non-PD group, including 6 cases with Parkinson-plus syndrome, 8 cases with DLB and 21 cases with other forms of parkinsonism.

### 3.2. Comparisons of Semi-Quantitative Measurements and ANN Classifier

**Figure 3:**
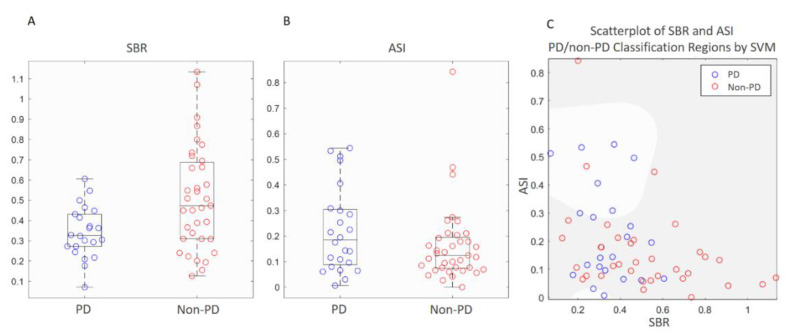
Distribution of indicators derived from semi-quantitative methods (SBR and ASI) in the test dataset (n = 57). (**A**) Dot diagram overlaid whisker-boxplot of SBR showed a wider range of distribution in the non-PD group. The range of the PD group almost totally overlapped with that of the non-PD group. (**B**) Dot diagram overlaid whisker-boxplot of ASI showed a wider range of distribution in the PD group. The range of the non-PD group almost totally overlapped with that of the PD group. (**C**) Scatterplot of SBR and ASI of both groups showing the classification results of median Gaussian kernel SVM with PCA. In the PD (lighter) region only one non-PD point was included, while there were 12 PD points in the non-PD’s (darker) region. The overall accuracy was 68.4% using this machine-learning method.

**Figure 4:**
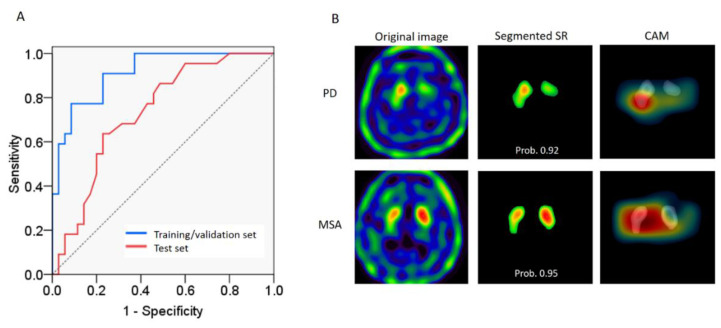
Classification of the test dataset using the ANN classifier. (**A**) The area under the receiver operating characteristic curve (AUROC) was 0.94 in the training/validation dataset (blue line) and 0.76 in the test dataset (red line). (**B**) Examples of classification using the ANN classifier for each group. Upper row is an example of PD and the lower row non-PDs. Left column: the images before scalp-mask and segmentation. Middle column: the images of segmented SR using the active contour model. Right column: the CAM represented with a heat map. The computer-vision weighted more on areas with a warmer color when examining the images. Overlaying on SR images showed that the computer focused most on the putamen. This PD subject was a 58-year-old male with symptoms of resting tremors in his right hand for 2 years and H&Y stage 2 when DAT-SPECT was obtained. Another example case of multiple system atrophy was a 69-year-old male who had symptoms of urinary incontinence, orthostatic hypotension, and cerebellar features of dysmetria and parkinsonism. The disease duration before DAT-SPECT was obtained was 2 years. Prob., probability of class.

The performances of classifying the test dataset using semi-quantitative measurements and ANN classifier were compared. The distributions of both SBR and ASI of the test dataset were found to be normal according to the Shapiro–Wilk test. The unpaired t tests between the PD and non-PD groups were *p* = 0.003 for SBR and *p* = 0.083 for ASI. The test datasets were classified using SBR and ASI, respectively. According to the boxplot, the distributions of SBR and ASI values between groups greatly overlapped (Figure 3A,B). Classification by SVM using features from the combination of SBR and ASI revealed that moderate Gaussian kernel through PCA feature extraction resulted in the best result among the methods of machine learning (Figure 3C). There were still several remarkable errors within each classification region. The classification accuracy was 68.4% with sensitivity and specificity of 31.8% and 91.4%, respectively, in predicting PD. For the ANN classifier, an accuracy of 92% was obtained through repetitively fine-tuning and validating the training dataset. Classification of the test dataset through best parameters (feature maps) from computer-vision with ANN revealed an accuracy of 86% with sensitivity and specificity of 81.8% and 88.6%, respectively, in predicting PD (Table 2). The performance of this classifier was favorable (Table 3). The AUROC was 0.94 for the training/validation dataset and 0.76 for the test dataset (Figure 4A). Another ANN classifier trained and tested using whole-brain images (without segmentation) from the same groups of subjects had lower accuracy, sensitivity, and specificity (Table 2).

### 3.3. Visualization of Computer-Vision through CAM

Class-activation mapping revealed the most discriminative parts of the images, and the results showed that computer-vision focused on the most informative regions of both sides of the putamen (tail of comma) (Figure 4B) to classify PD and non-PD. However, which of the intensity, shape, curvature, or convexity of contour was the most characteristic feature was not available for further analysis.

## 4. Discussion

The accuracy of differentiating parkinsonian syndromes through visually rating DAT-SPECT images has been reported to be quite low [29]. Although the semi-quantitative measurements revealed statistical differences between the PD and non-PD groups in testing data in this study, the individual values overlapped greatly between groups (Figure 3). The ANN classifier provided a higher specificity in prediction using “computer-vision parameters”. Our results showed acceptable accuracy in differentiating PD from parkinsonism caused by other disorders using only DAT-SPECT images without additional information. The performance of the ANN classifier, with sensitivity and specificity both above 80%, was comparable to that of quantitative olfactory examinations and MIBG myocardiac scintigraphy suggested by diagnostic guidelines. Furthermore, this method is promising because of several advantages: (1) as the sample size of the dataset increases, training results can be further improved; (2) with an adequate number of images taken during the earlier phase of disease (PD or atypical parkinsonian syndromes), the ANN classifier may be trained to identify PD at an early phase [24] or even possibly at a preclinical phase; (3) medical centers and hospitals can train a site-specific ANN classifier using SPECT images based on their own existing dataset without developing new diagnostic modalities or purchasing expensive machines, especially for places where MIBG is not available; (4) SPECT is more widely available, so that when the diagnosis is not straightforward, physicians tend to order SPECT imaging first to confirm striatal neuron loss, such as to differentiate essential tremors from PD, but not MIBG myocardial scintigraphy before proving a neurodegenerative disease in the early phase; and (5) PD can be differentiated from many disease types of parkinsonism, not just a few Parkinson-plus syndromes or other Lewy body diseases [19,30]. Therefore, this classifier is more applicable when facing uncertain types of parkinsonism in clinical practice. In addition, we chose easily-accessible methods and basic application programs, including an active contour model for segmentation and AlexNet for learning and classification. These two tools are widely utilized and can be obtain from online resources. All the processing in this paper were done by a PhD student with entry-level graphics processing unit (GPU, NVIDIA GeForce GTX 1060) in a personal computer. This avoided the need for complicated image processing procedures, experienced engineers, or high-performance computer equipment.

For a feasible classifier, the discriminative parameters do not necessarily need to be clinically correlated, such as extracted features from component analysis [25] or shape/morphological fitting characteristics [18]. Even though SPECT is an imaging technique with a lower resolution than MRI, ANN analyzes an image by decomposing hundreds of thousands of pixels into hundreds of pixeled “matrices” to extract local features. Computer-vision sees patterns of relationships between decomposed pixels of matrices, even if the images do not represent actual anatomical structure in fine detail. However, the excessive number of parameters is also a pitfall of ANN. When training the neural network with SPECT images of whole-brain uptake, the accuracy was lower. This might be because the ANN automatically counted differences in intra- and extra-striatal uptake or background noise equally. Unlike computer-vision, when humans examine DAT-SPECT images they spontaneously focus on the uptake in the SR much more than in extra-striatal regions. This has been shown in previous studies in which better classification accuracy was achieved by looking only at the SR rather than at the whole brain [20]. It could be argued that comparing only the SR may result in the loss of too much information. For example, PD, multiple system atrophy, and DLB are all associated with the same fibrillar α-synuclein protein, but the differences are the sites in which it accumulates in the brain. Although it may be reasonable to compare different patterns of the whole brain, according to prior studies, only the SR was sufficient to differentiate PD from multiple-system atrophy or DLB [21,31].

In order to feed the ANN with segmented images, an active contour model is not only a feasible tool to select the ROI of the SR as with human vision, but also a highly-reproducible method to diminish inter-individual errors in ROI contour outlining. The successful classification using a combination of active contour method and ANN was supported by CAM. The most informative area to differentiate PD from parkinsonism caused by other disorders was the putamen. The region on which computer-vision focused most in this study has also been reported in previous studies using semi-quantitative measurements and other imaging modalities such as diffusion MRI.

We proposed a feasible method to develop a diagnostic tool capable of differentiating PD from parkinsonism caused by other disorders at an early stage through DAT-SPECT images. However, there are some limitations: (1) As a general rule, a bigger dataset is better for training an ANN. A test dataset with more cases with a confirmed diagnosis or even a prospective study is needed to prove and improve the accuracy. Unfortunately, the number of medical images is usually limited. In this study, we used learning from a well-pretrained network to address this limitation. To develop customized and appropriate layers of a neural network is another solution [24,28] to avoiding overfitting during training. (2) Using images from multi-centers to recruit a larger amount of data may result in compatibility problems among different reconstruction algorithms and different machines. Although ANNs may accommodate discrepancies resulting from different reconstruction algorithms by using more parameters, the accuracy may be lower. To consider raw image information such as a “probability map” before reconstruction, appropriate normalization protocols may also be able to solve this issue [25]. (3) In the clinical scenario, the really difficult cases are those that did not fit any diagnostic criteria, the so called gray cases. Although this ANN classifier was trained by images from subjects with discriminative features, it had the potential to study the diagnostic accuracy in gray cases. However, the exact diagnosis of these gray cases is the main obstacle and may depend on pathology. (4) The basis for the diagnosis in this study was purely clinical without underlying pathology. (5) AlexNet is not the most up-to-date tool. To further explore the methodology of applying a pretrained neural network, advanced ANN with more convincing validation algorithms should be considered. (6) Differential diagnosis based only on images could be limited. To promote diagnostic accuracy, a combination of clinical, neuroimaging, and neuropsychology may provide better discrimination between parkinsonisms [23].

## 5. Conclusions

In this study, an ANN classifier focusing on the putamen region of DAT-SPECT images outperformed the classical biomarkers to differentiate PD from parkinsonism caused by other disorders, with an accuracy of 86% (sensitivity of 81.8% and sensitivity of 88.6%). This method is easily accessible and clinically applicable and provides opportunities to develop an early diagnostic tool to allow for the appropriate application of disease-modifying therapies, in clinical trials and even possibly for bedside treatment in the future.

## Figures and Tables

**Table 1:** Demographic characteristics of the subjects.

Data	Training/Validation Set (*n* = 205)			Test Set (*n* = 57)		
Group	PD	Non-PD	*p* Value	PD	Non-PD	*p* Value
Age (years) (mean ± SD)	65.4 ± 10.2	66.6 ± 12.8	0.44	70.3 ± 9.8	70.6 ± 13.4	0.93
Gender (F/M)	52/53	45/55	0.51	8/14	12/23	0.87
Mean disease duration (years) (IQR)	2.32 (2)	1.89 (1)	0.27	2.57 (2.5)	3.56 (3)	0.34

PD, Parkinson’s disease; SD, standard deviation; IQR, interquartile range.

**Table 2:** Comparisons of the prediction accuracy of the test dataset with different classifiers.

Classifier	SVM	ANN	
Learning Method	Machine Learning	Deep Learning	
**Input data**	SBR & ASI	Whole-brain image	SR image
**Accuracy**	68.4%	68.4%	86.0%
**Sensitivity**	31.8%	81.8%	81.8%
**Specificity**	91.4%	60.0%	88.6%

SVM, support vector machine; ANN, artificial neural network; SBR, specific binding ratio; ASI, asymmetry index; SR, striatal region.

**Table 3:** Confusion matrix of ANN classifier for predicting PD.

	Predicted Positive (Classified as PD)	Predicted Negative (Classified as non-PD)	
Actual positive (PDs = 22)	TP 18	FN 4	Sensitivity (recall) 0.818
Actual negative (non-PDs = 35)	FP 4	TN 31	Specificity 0.886
	Precision 0.818	Negative Predictive value 0.886	Accuracy 0.860
	**F1 score**: 2 × (precision × recall)/(precision + recall) = **0.818**		

PD, Parkinson’s disease; TP, true positive; FP, false positive; FN, false negative; TN, true positive.

## Data Availability

Data sharing not applicable.

## Appendix A

### DAT-SPECT Scan and Reconstruction Protocol

Subjects were intravenously administered with 740 MBq (20 mCi) (99mTc) TRODAT-1 (a radiolabeled form of a tropan derivative for the selective labeling of DAT) in a quiet environment about 10 min after insertion of an intravenous line. The SPECT data were obtained using an energy window of 15% centered on 140 keV for (99mTc). Imaging of (99mTc) TRODAT-1 was initiated approximately 240 min after injection, and SPECT images were acquired over a circular 360° rotation in 120 steps, 50 s per step, in a 128 × 128 × 16 matrix. The images were then reconstructed using Butterworth and Ramp filters (cutoff frequency = 0.3 Nyquist, and power factor = 7) with attenuations according to Chang’s method [1], and the reconstructed transverse images were realigned parallel to the canthomeatal line. The slice thickness of each transverse image was 2.89 mm [1]. Chang LT. A method for attenuation correction in radionuclide computed tomography. IEEE Trans Nucl Sci. (25) (1978) 638-43.
